# Unique Organizational and Functional Features of the Cytochrome *c* Maturation System in *Shewanella oneidensis*

**DOI:** 10.1371/journal.pone.0075610

**Published:** 2013-09-10

**Authors:** Miao Jin, Yaoming Jiang, Linlin Sun, Jianhua Yin, Huihui Fu, Genfu Wu, Haichun Gao

**Affiliations:** College of Life Sciences and Institute of Microbiology, Zhejiang University, Hangzhou, Zhejiang, China; Instituto de Tecnologia Quimica e Biologica, Portugal

## Abstract

*Shewanella* are renowned for their ability to respire on a wide range of electron acceptors, which has been partially accredited to the presence of a large number of the *c*-type cytochromes. In the model species *S. oneidensis* MR-1, at least 41 genes encode *c*-type cytochromes that are predicted to be intact, thereby likely functional. Previously, in-frame deletion mutants for 36 of these genes were obtained and characterized. In this study, first we completed the construction of an entire set of *c*-type cytochrome mutants utilizing a newly developed *att*-based mutagenesis approach, which is more effective and efficient than the approach used previously by circumventing the conventional cloning. Second, we investigated the cytochrome c maturation (Ccm) system in *S. oneidensis*. There are two loci predicted to encode components of the Ccm system, *SO0259-SO0269* and *SO0476-SO0478*. The former is proven essential for cytochrome *c* maturation whereas the latter is dispensable. Unlike the single operon organization observed in other γ-proteobacteria, genes at the *SO0259-SO0269* locus are uniquely organized into four operons, *ccmABCDE*, *scyA*, *SO0265*, and *ccmFGH-SO0269*. Functional analysis revealed that the *SO0265* gene rather than the *scyA* and *SO0269* genes are relevant to cytochrome *c* maturation.

## Introduction

Cytochromes, proteins carrying the heme group as prosthetic cofactor, can be classified into *a*-, *b*-, *c*-, *d*-, and o-types depending on the variations on the protoporphyrin ring [1]. Cytochromes of *c*-type, existing as membrane-bound proteins or soluble periplasmic proteins, play vital roles in bacterial respiration and photosynthesis as enzymes to exchange electrons with the bound substrates or as pure electron carriers to shuttle electrons. It is estimated that one third of cytochrome hemes are located at enzyme active sites while the rest are redox cofactors transporting electrons in an electron transfer chain [1–3]. The unique feature of *c*-type cytochromes is the covalent attachment of the cofactor to the protein polypeptide at the cysteines within the signature haem *c* binding motif (HBM) CX _n_CH (X stands for any amino acid, n = 3, 4, 15) [1–3]. Although the motif is well conserved across species, the sequences A/FX _2_CH have been described as HBMs in bacterial or mitochondrial proteins [4,5].

*Shewanella oneidensis* MR-1, a facultative Gram-negative anaerobe, is renowned for its remarkable anaerobic respiration ability. Linked to this unique characteristic is a high cytochrome content, especially *c*-type [6,7]. Compared to *Escherichia coli* which hosts only 5~7 *c*-type cytochromes, *S. oneidensis* is predicted to possess as many as 44 *c*-type cytochrome proteins by screening for the canonical HBMs in the proteome [8–10]. Although a few appear to be degenerated due to frameshift mutations within the coding sequence [9], the number of *c*-type cytochromes in *S. oneidensis* may increase with time as proteins with the non-canonical HBMs may be found. For example, haem *c* group II in OTR (SO4144, octaheme tetrathionate reductase) is ligated to C_74_X_75_X_76_C_77_H_78_ and a lysine residue (Lys56), which are in proximity structurally [11,12].

Extensive biochemical and genetic investigations have revealed that three systems predominate in *c*-type cytochrome maturation although some specialized ones have been identified in recent years [1,13,14]. The definition of the three systems is based on the presence of specific assembly components that are unique to each maturation system. System I, also called Ccm (cytochrome c maturation), extensively studied in α and γ-proteobacterial models, is composed of up to 12 components, CcmA to CcmH, CcmI, DsbA, DsbB, and DsbD/CcdA. As a γ-proteobacterium, *S. oneidensis* is predicted to have system I as it encodes analogues to CcmC, CcmF, and CcmE, the signature components for this system [1,6]. However, this organism differs from other γ-proteobacteria in *ccm* gene organization significantly [15]. Unlike the common pattern that all *ccm* genes are clustered together and transcribed in the same orientation, in *S. oneidensis* two genes separate *ccmABCDE* from *ccmFGH*, resulting in two ccm operons, which are transcribed divergently. In addition, it is interesting to note that this microorganism has a second *ccmF* gene located elsewhere on the chromosome.

In *S. oneidensis*, the functionally defined *c*-type cytochromes mostly are terminal reductases as their corresponding deletion mutants display distinguishable phenotypes, such as NrfA, NapB (small subunit of nitrate reductase), FccA (fumarate reductase), and DmsE (subunit of dimethyl sulfoxide (DMSO) reductase), to name a few [13–15]. Furthermore, those involved in respiration of insoluble electron acceptors are relatively better understood because the subject has been under intensive investigation for nearly three decades [7,16]. To facilitate systematic characterization of *c*-type cytochromes, we endeavored to construct a whole set of single-gene knockouts but failed with five of them in our previous study [10]. Here, we first developed an *att*-based mutagenesis approach and completed the construction of an entire set of *c*-type cytochrome mutants with it. This new approach bypasses the conventional cloning step, which reduces the effectiveness and efficiency of the system used before. In addition, we examined the Ccm system for determination of the essential components of the system. More intriguing, this research identifies a protein encoded in the *ccm* locus showing varying degrees of essentialness for respiration of different electron acceptors.

## Results

### Activeness of *c*-type cytochrome genes at transcriptional levels

Recently, proteomic measurements reveal 23 of all *S. oneidensis c*-type cytochromes in cells grown with ferric citrate (soluble Fe(III)) or MnO_2_ (insoluble Mn(IV)) as electron acceptors under anaerobic conditions. This observation implicates that production of a considerable number of *c*-type cytochromes in physiologically relevant amounts is condition-specific [17]. Such information not only provides insights into their physiological functions but also may help to understand technological difficulties encountered during construction of a whole set of *c*-type cytochrome single-gene mutants. In our previous study, we failed to generate in-frame deletions for *scyA* (*SO0264*), *torC* (*SO1233*), *SO1748*, *ccpA* (*SO2178*) or *SO3056* after multiple attempts [10].

**Figure 1:**
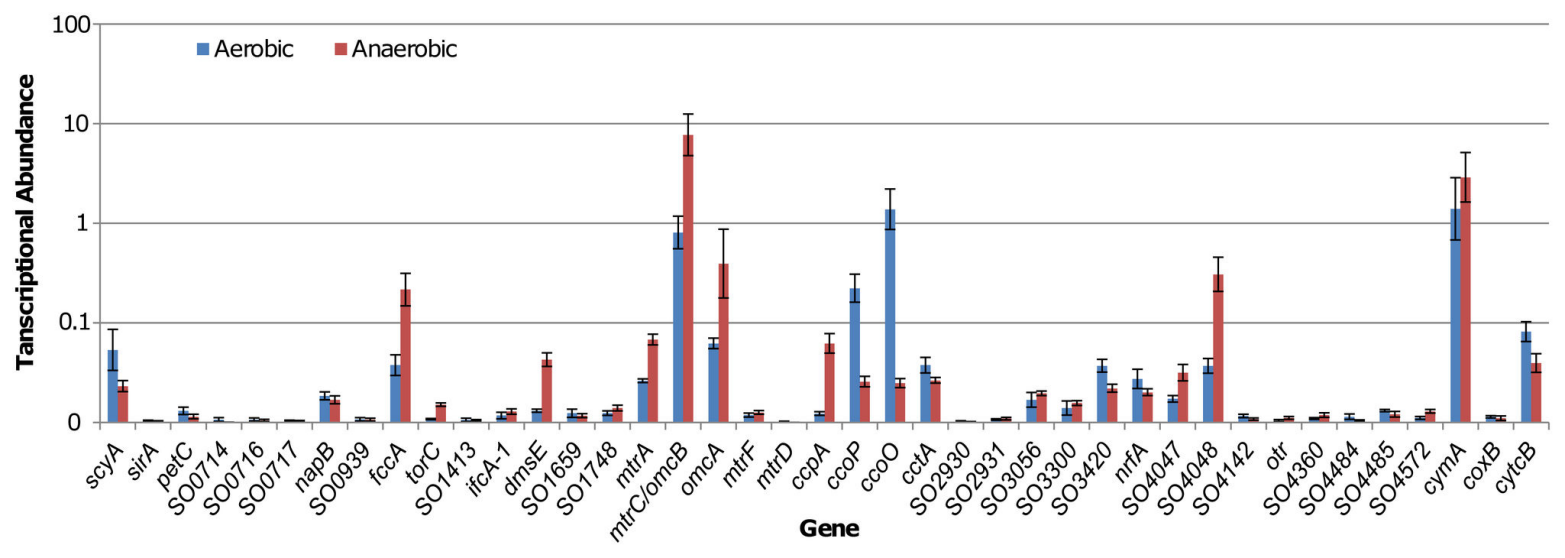
Expression of genes encoding *c*-type cytochromes in *S. oneidensis*. qRT-PCR analysis of RNA extracted from mid-logarithmic growing cells (OD_600_, ~0.4 and ~0.15 for aerobic and anaerobic cultures respectively). All data were normalized to expression of the *arcA* and *recA* genes, which were constant during the exponential growth phase. Numbers reported are standardized to expression of the *arcA* gene, but the same trends are observed when standardized to expression of the *recA* gene. Error bars represent standard deviation for triplicate cultures. *SO1778* is displayed as *mtrC*/*omcB* because both names have been used in previous publications, but only *mtrC* is used in the rest of this article since it is accepted more widely.

To understand expression characteristics of *c*-type cytochromes in cells grown under aerobic and anaerobic conditions, we examined the mRNA abundance of their coding genes in exponentially growing cells using quantitative reverse transcription PCR (qRT-PCR) (Figure 1). The *cymA* gene, as reported repeatedly [18,19], showed a constant high level of transcription that is oxygen-independent. This is not surprising because the protein plays a key role in mediating electron transport in multiple respiratory pathways [20,21]. Interestingly, impacts of oxygen on transcription of the major components of the metal reduction pathway *mtrA*, *mtrC*, and *omcA* were also negligible. Combining growth defects of the ∆*mtrA* and ∆*mtrC* strains under aerobic conditions [10], these data suggest that these proteins and/or the pathway may be implicated in other physiological processes of general importance. Consistent with findings that the *cbb*_3_ oxidase plays a predominant role in oxygen respiration [22], both *ccoP* and *ccoO* (encoding two essential subunits of the *cbb*_3_ oxidase) were transcribed at significantly higher levels in cultures grown with oxygen than in those without oxygen. Other genes displaying the same pattern include *scyA*, *cctA*, and *cytcB*, implicating that these small electron shuttling *c*-type cytochromes may be more likely to function under aerobic conditions. In contrast, *fccA* (fumarate reductase) and *dmsE* (subunit of the DMSO reductase) were transcribed at higher levels in anaerobic than aerobic cultures. It is worth mentioning that transcription of a number of genes, most of which encode proteins of unknown function, was extremely low regardless of growth conditions. While one of possible explanations is that the culturing conditions do not favor transcription of these genes, at least one alternative has been reported. In *S. oneidensis*, the *caa*_3_ oxidase has a negligible role in oxygen respiration because its coding operon (including *coxB*) is not expressed to a physiologically relevant level [22].

### Development of an *att*-based mutagenesis system and generation of a complete set of *S. oneidensis* *c*-type cytochrome mutants

**Figure 2:**
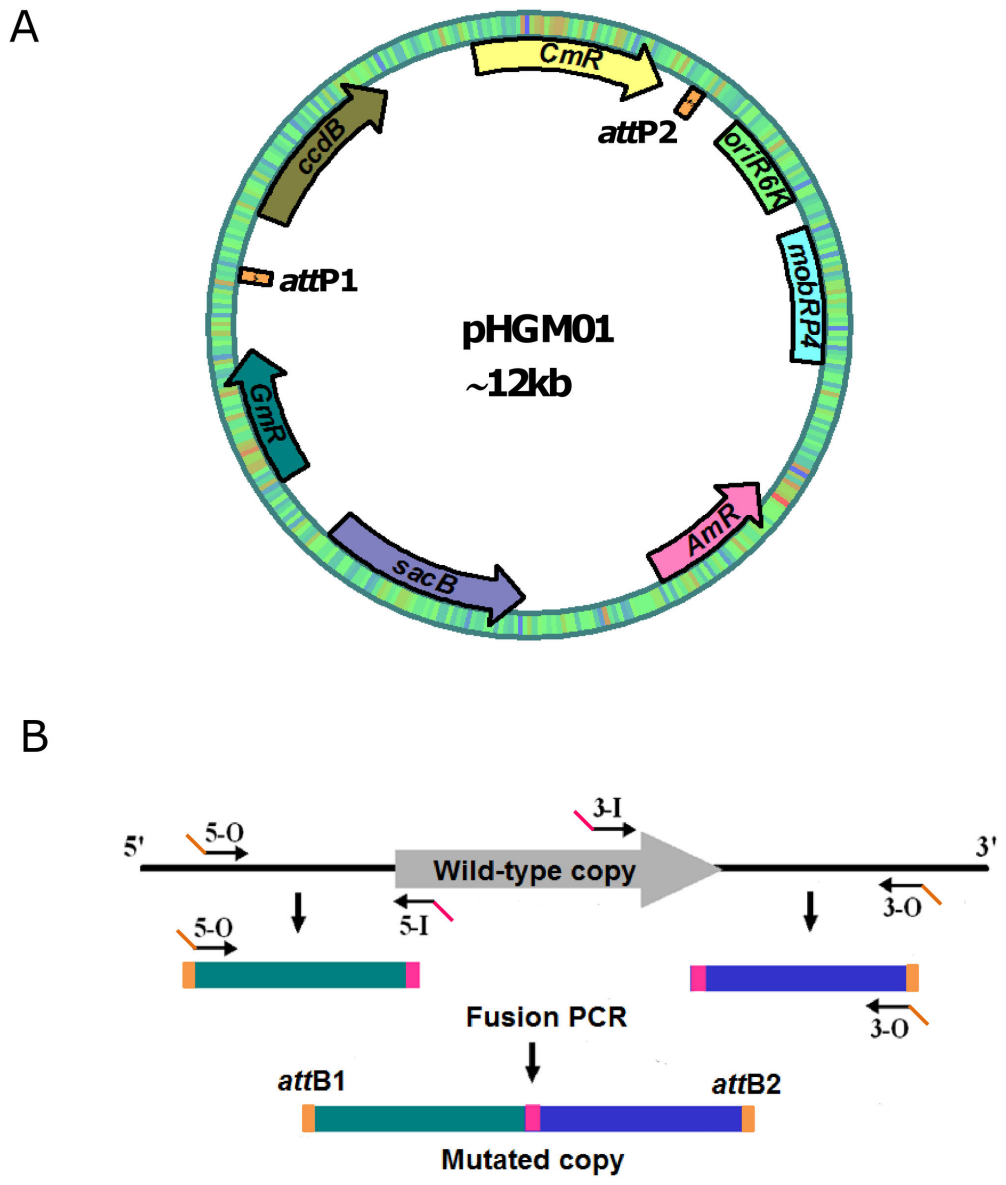
The *att*-based mutagenesis developed in this study. **A**. Mutational construct delivery vector pHGM01. The vector was created by introducing into the *Sac*I site of pDS3.0 a fragment containing *att*P1, *ccdB*, Cm^R^, and *att*P2 amplified from pMK2010. **B**. Generation of a fusion PCR product for BP recombination. Outside primers 5-O and 3-O contain *att*B1 and *att*B2 sequences, respectively. Inside primers 5-I and 3-I contain linking sequences respectively, which are complementary to each other.

In *S. oneidensis*, in-frame single-gene deletion strains were successfully constructed for 36 out of 41 *c*-type cytochrome genes using either the fusion-PCR- or *cre-lox*-based mutagenesis approach [10]. Among five *c*-type cytochrome genes that we failed to remove, only *scyA* was highly expressed aerobically as shown above, a condition under which the previous attempts are made [10], suggesting that most of these genes may not play an important role in aerobiosis. In addition, these genes do not carry common characteristics in sequences and possible secondary structures that may interfere with PCR amplification or recombination. As a consequence, the failure is likely ascribable to the mutagenesis approaches. Indeed, both the fusion-PCR-based and *cre-lox*-based methods proved inefficient and sometimes even ineffective because of multiple rounds of PCR and conventional cloning. Additionally, the mutagenesis delivery vector pDS3.0 is rather large (^~^10 kb), resulting in few unique restriction enzyme sites available for cloning, especially for large open reading frames (ORFs). We therefore sought to develop a new mutagenesis system free of conventional cloning for *Shewanella* and other organisms in which plasmids with a pR6K origin (*ori*_*R6K*_) could not replicate. A PCR fragment containing bacteriophage lambda *att*P sequences bracketing the Cm^R^ cassette and toxic gene *ccdB*, generated using pMK2010 as the template, was introduced into pDS3.0, resulting in pHGM01 [23] (Figure 2A). To produce an in-frame deletion construct for ORF of interest, two PCR fragments flanking the ORF were generated using primers containing the *att*B sequences (outside primer) and linking sequences (inside primers) with the genomic DNA as the template by PCR and then joined together by the second round of PCR with primers containing the *att*B sequences (fusion PCR step) (Figure 2B). The resulting fusion PCR product was transferred into pHGM01 by lambda BP recombination. It is worth noting that the recombination also occurred effectively in the presence of unwanted smaller DNA fragments, which are common, often inevitable, byproducts at the fusion PCR step and regarded to be the major obstacle for conventional cloning. The correct mutagenesis vector, verified by DNA sequencing, was then transferred into *S. oneidensis* by conjugation for the subsequent steps of the fusion-PCR-based mutagenesis procedure [24].

With this new system, we obtained in-frame deletion strains for the *scyA*, *torC*, *SO1748*, *ccpA*, and *SO3056* genes. Physiological characterization of these five mutants was then carried out to assess impacts of each mutation as previously described [10]. To support growth, oxygen or one of following chemical agents was used as the sole electron acceptor: DMSO, fumarate, trimethylamine N-oxide (TMAO), NaNO_3_, Fe-Citrate, and MnO_2_ under anaerobic conditions. Results showed that none of mutations had a statistically significant effect on growth under any test condition except for the ∆*torC* strain grown on TMAO (Table 1). Consistent with the essentiality of the *torECAD* operon for TMAO reductase activity [25], the loss of the *torC* gene prevented *S. oneidensis* from growing on TMAO.

**Table 1:** Physiological characterization of mutants constructed in this study ^a^.

Mutant	Possible function of deleted gene	DMSO	Fumarate	TMAO	NaNO_3_	Fe-Citrate	MnO_2_	O_2_
HG0264	Periplasmic monoheme cytochrome *c*_5_	+	+	+	+	+	+	+
HG0265	Cytochrome *c* biogenesis protein CcmI	**--**	**--**	+	**--**	**--**	**--**	+
HG0266	Cytochrome *c* biogenesis protein CcmF	**─**	**─**	**─**	**─**	**--**	**--**	**--**
HG0268	Cytochrome *c* biogenesis protein CcmH	**─**	**─**	**─**	**─**	**--**	**--**	**--**
HG0269	Unknown	+	+	+	+	+	**+**	+
HG0478	Cytochrome *c* biogenesis protein CcmF2	+	+	+	+	+	+	**--**
HG0478-6	CcmF2-NrfF-SO0476, Unknown	+	+	+	+	+	**+**	+
HG1233	TMAO reduction TorC	+	+	**─**	+	+	+	+
HG1748	Periplasmic monoheme cytochrome *c*	+	+	+	+	+	+	+
HG2178	Cytochrome *c*_5_ peroxidase CcpA	+	+	+	+	+	+	+
HG3056	Tetraheme cytochrome *c*	+	+	+	+	+	+	+

^a^Growth of the wild type and mutant strains was monitored in M1 medium with lactate as electron donor and one of listed chemicals as electron acceptor. Values (growth rate and/or maximum cell density) of mutant strains were normalized to that of the wild type: +, > 75%; --, between 75% and 25%; -: < 25%. Experiments were performed at least three times and standard deviation was less than 10% of values.

### *In silico* analysis of Ccm system of *S. oneidensis*

**Figure 3:**
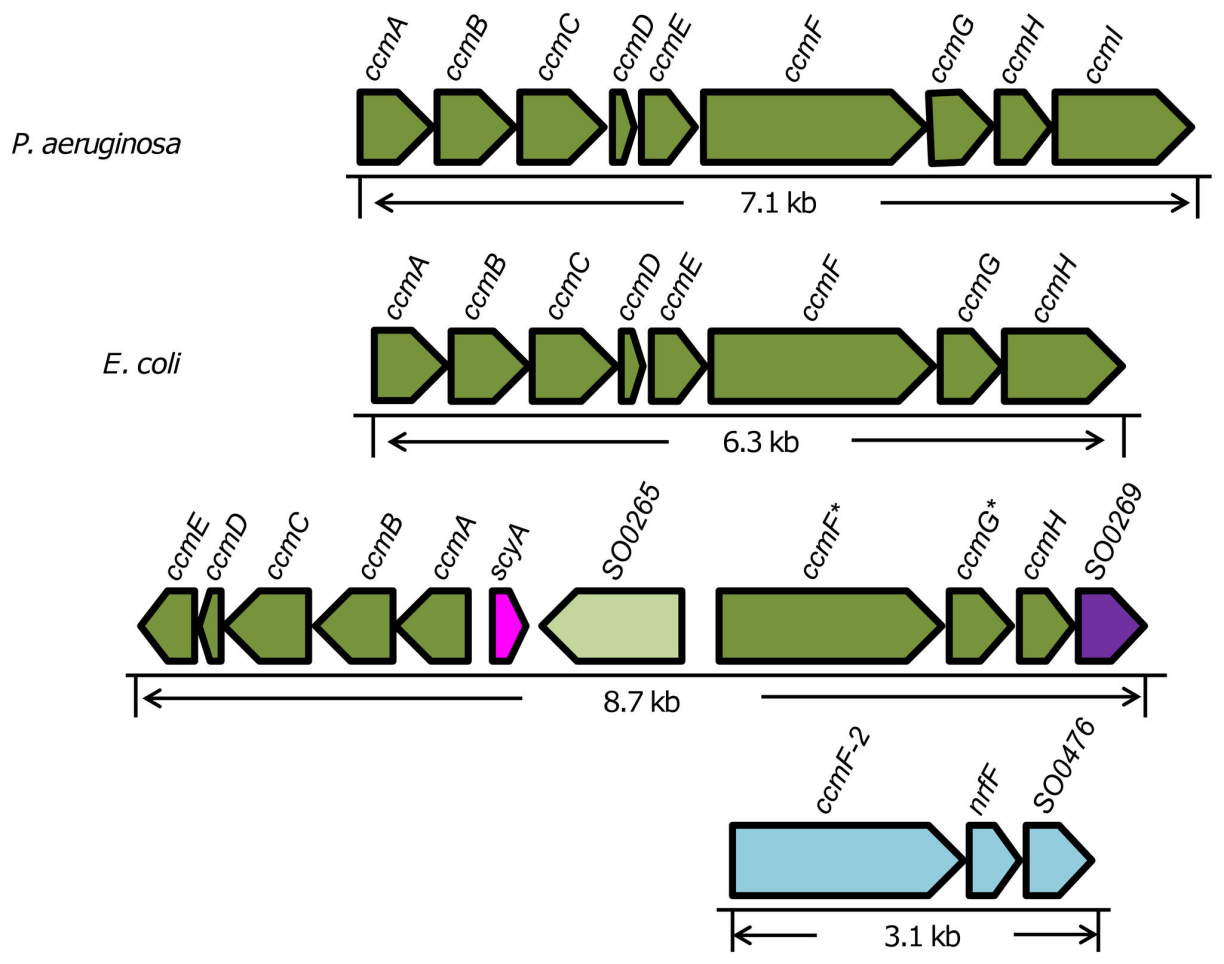
Organization of the *ccm* genes in γ-proteobacteria. The most common gene arrangement is represented by *Pseudomonas aeruginosa*, in which CcmH and CcmI are separate from each other. The less common one is represented by *E. coli*, whose CcmH is a fusion protein between CcmH and the C-terminal portion of CcmI found in other bacteria. In *S. oneidensis*, two loci for predicted *ccm* genes were shown. Genes renamed in this study were labeled with an asterisk mark. Genes are drawn to scale. The data were from http://img.jgi.doe.gov/cgi-bin/w/main.cgi.

*S. oneidensis* is distinct from other γ-proteobacteria in organization of the *ccm* genes (Figure 3). According to the genome annotation, *S. oneidensis* has two loci for *ccm* genes, *SO0259-0269* and *SO0476-0478*. The first locus includes operons *ccmABCDE*, *scyA*, *SO0265*, and *ccmF*_*1*_ *-dsbE-ccmH-SO0269*. The *ccmABCDE* operon exists as a single copy in all 27 sequenced *Shewanella* and the essentiality of their products for cytochrome *c* maturation have been firmly established [1–3]. Within the *ccmF*_*1*_ *-dsbE-ccmH-SO0269* operon, the *dsbE* gene (*SO0267*) has been proposed to encode a real CcmG protein, whose homologues are implicated in the reduction of disulphide bonds of the apocytochrome *c* prior to haem ligation in a variety of bacteria [15]. This proposal is supported by sequence analysis. SO0267 shares a highest sequence similarity with well-characterized *E. coli* CcmG proteins: 50%/72% (identity/positive), with an expect value of 2e^-38^. We therefore renamed *dsbE* as *ccmG*, rendering a syntenic consistency to the *ccmF *_*1*_ *GH-SO2069* operon as the order of the *ccmFGH*(*I*) genes are perfectly preserved in γ-proteobacteria. However, the last gene of the *ccmF *_*1*_ *GH-SO0269* operon is a mystery. Although the *E. coli* CcmH_EC_ is 350 amino acids (aa) in length, a size equivalent to a combination of *S. oneidensis* CcmH (159 aa) and SO0269 (194 aa), SO0269 shows a modest sequence similarity to CcmG_EC_ (32%/52%, 3e^-8^), but not to CcmH_EC_. In addition, SO0269 has no sequence similarity to *P. aeruginosa* CcmI_PA_ (407 aa). Moreover, an analogue of *SO0269* is completely missing from two sequenced *Shewanella* strains, *S* *. denitrificans* OS217 and *S. violacea* DSS12, implying that SO0269 may not be required for cytochrome *c* maturation. Two remaining genes in this locus are *scyA* and *SO0265*. It is interesting to note that SO0265 (415 aa) is a homologue of CcmI_PA_ with sequence similarity of 33%/54% and expect value of 2e^-37^, implicating a possible role for this protein in cytochrome *c* maturation.

The second locus consists of a single operon, *ccmF*_*2*_ *-nrfF-SO0476*, which is perfectly preserved in all sequenced *Shewanella* . Like CcmF_1_, CcmF_2_ is highly homologous to CcmF_EC_ with sequence similarity of 40%/60% and expect value of 2e^-137^. Such a high level of sequence conservation strongly suggests a close evolutionary relationship between the two proteins. Moreover, NrfF (SO0477) and SO0476 are homologues of CcmH (37%/69%, 2e^-16^) and SO0269 (38%/63%, 3e^-37^), respectively.

### Maturation of *c*-type cytochromes is independent of *ccmF_2_-nrfF-SO0476*

**Figure 4:**
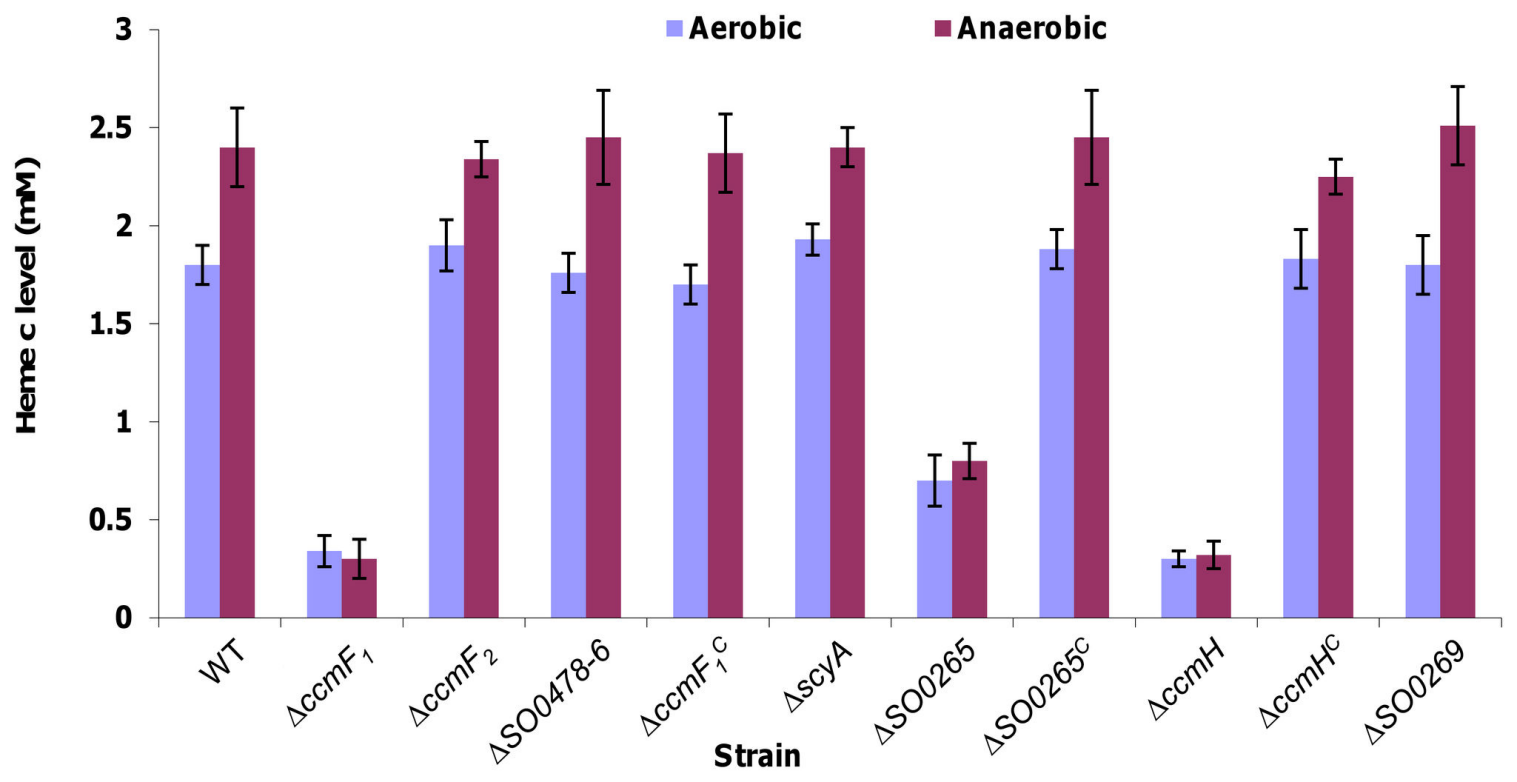
Heme *c* levels in *S. oneidensis* strains. wild type, ∆*ccmF1*, and ∆*ccmF2* strains. Mid-logarithmic growing cells (OD_600_, ^~^0.4 and ^~^0.15 under aerobic and anaerobic conditions respectively) were collected for the heme *c* assay. ∆*ccmF*_*1*_^*c*^ and ∆*SO0265* ^*c*^ represents the mutants carrying pHG102-*ccmF*_*1*_ and pHG102-*SO0265*, respectively. All other strains carry empty vector pHG102. Three independently collected samples were assayed and the averaged levels were presented in mM per g of proteins. Error bars represent the standard deviations of the data.

The *in silico* analysis raised questions about the role played by two CcmF components as well as uncertain SO0265 and SO0269 in *S. oneidensis*. To evaluate the impact of these proteins on cytochrome *c* maturation, we constructed mutants defective in one of their coding genes. Deletion of *ccmF*_*2*_ did not elicit any difference from the parental wild type strain but the loss of *ccmF*_*1*_ resulted in light-colored colonies, an indicator for the reduced amount of *c*-type cytochromes. Quantification of intracellular heme *c* content confirmed that the ∆*ccmF*_*1*_ strain was severely impaired in heme *c* production whereas the ∆*ccmF*_*2*_ strain had a heme level identical to that of the wild type (Figure 4). Importantly, the deficient in heme *c* resulted from the *ccmF1* deletion was corrected by its expression *in trans*, indicating that the phenotype observed was due to the intended mutation.

To assess impact of loss of the *ccmF*_*1*_ and *ccmF*_*2*_ genes on respiration of various electron acceptors, the wild type and mutant strains were inoculated into M1 medium supplemented with one of tested electron acceptors and growth was monitored. With all tested electron acceptors, the wild type and ∆*ccmF*_*2*_ strains were indistinguishable but the ∆*ccmF*_*1*_ strain was distinct (Table 1). In the absence of CcmF_1_, the bacterium lost ability to grow on DMSO, fumarate, TMAO, or NaNO_3_ completely but was still capable of growing on oxygen, Fe-Citrate, and MnO_2_, albeit significantly impaired. To examine impact of the entire *ccmF*_*2*_ *-nrfF-SO0476* operon on cytochrome *c* maturation, we removed all of these three genes from the wild type and characterized the resulting mutants as described above. Similar results were obtained compared to the ∆*ccmF*_*2*_ strain. These data, collectively, indicate that the *ccmF*_*2*_ *-nrfF-SO0476* operon is not involved in cytochrome *c* maturation in *S. oneidensis*. We, therefore, renamed the *ccmF*_*1*_ gene as *ccmF*.

### Maturation of *c*-type cytochromes requires SO0265 and CcmH but not SO0269

The *scyA* gene locates in the middle of the *ccm* cluster and has been suggested to be involved in cytochrome *c* maturation [19]. However, our data from the physiological characterization as shown above imply that the protein may not have a role in the process, at least not significantly. This notion is further supported by that the ∆*scyA* strain had similar levels of heme *c* production compared to the wild type (Figure 4).

We then made an attempt to determine whether three remaining genes in the cluster, *SO0265*, *ccmH*, and *SO0269*, are relevant to cytochrome *c* maturation in *S. oneidensis*. As shown above, the *ccmH* gene of *S. oneidensis* is much shorter than its *E. coli* counterpart. Therefore, whether this protein is essential for cytochrome *c* maturation merits an investigation. Interestingly, phenotypes resulting from deletions of these genes were different (Figure 4 and Table 1). Apparently, the *ccmH* gene is totally essential for the process evidenced by that its deletion resulted in a *ccmF* phenotype. Loss of the *SO0265* gene not only caused significant reduction in the bacterial growth on DMSO, fumarate, NaNO_3_, Fe-Citrate or MnO_2_, but also impaired growth with oxygen. However, relevance of the gene to TMAO respiration remained to be determined. In line with these growth phenotypes, the *SO0265* mutant produced heme *c* approximately 35% relative to the wild type strain. In contrast, deletion of the *SO0269* gene had little impact on aerobic and anaerobic growth as well as heme *c* production. As a result, whether this protein is part of the Ccm system in *S. oneidensis* remains to be determined.

## Discussion

A new in-frame deletion mutagenesis method, which is based on the site-specific recombination system used by phage λ to integrate its DNA in the *E. coli* chromosome, has been developed and successfully utilized in this study. To construct *Shewanella* mutants, fusion PCR technique with various suicide plasmids has been the most frequently utilized [10,24,26,27]. Because of the large size of suicide plasmids and unwanted PCR byproducts, these methods with traditional cut-ligation cloning suffers from the lack of unique restriction sites and/or a low efficiency for ligation of the fusion PCR products. By exploiting the site-specific recombination mechanism, our new system bypasses the need for restriction enzymes and ligation to introduce the fusion PCR products, substantially enhancing cloning efficiency.

The unique organization of the *ccm* genes is preserved in all sequenced *Shewanella* strains, implicating a common ancestral linkage. Despite a high level of sequence similarity with CcmF, CcmF_2_, as well as other proteins encoded by the *ccmF*_*2*_-*nrfF*-*SO0476* operon, has no role in cytochrome *c* maturation. Within the genuine *ccm* cluster, there are two puzzling genes, *SO0265* and *SO0269*. *S. oneidensis* has an NrfAH type of nitrite reduction system, in which the specific electron transport protein NrfH is replaced by CymA [18]. Given that all NrfA proteins contain an unconventional CXXCK haem-binding motif, which is recognized by a specific haem lyase as in *E. coli* and *Wolinella* *succinogenes* [28–30], it is conceivable that such a lyase exists in *S. oneidensis*. In a recent report, SO0265 has been suggested to be responsible for haem ligation to the atypical CXXCK site [31]. However, our data indicate that SO0265 certainly plays a more general and important role in cytochrome *c* maturation as the specific CXXCK lyases are not involved in haem ligation to the conventional sites [28–30]. Given that SO0265 is homologous to CcmI_PA_, it is likely that SO0265 is a functional equivalence of CcmI, which is currently under study. In the case of SO0269, a question needed to be addressed is whether the protein has a subtle role in cytochrome *c* maturation or is simply a redundant and degenerated copy of CcmG.

Recently, a few studies have been conducted to elucidate functions of *c*-type cytochromes that are poorly understood [17,32,33]. We now know that both CctA and FccA, two small abundant periplasmic *c*-type cytochromes, shuttle electrons from cytoplasmic membrane-bound CymA to outer-membrane-bound MtrA [32]. ScyA, also abundant in the periplasm, is proposed to be the electron donor to the dihaem CcpA, which functions as cytochrome *c* peroxidase to protect cells from oxidative damage under anaerobic conditions [33]. Together with our physiological analysis, it is apparent that ScyA functions more likely as an electron mediator than as a component of the Ccm system.

*Shewanella* , along with *Geobacter* , *Anaeromyxobacter* and *Desulfovibrio* , are examples of bacterial genera with a large number of predicted *c*-type cytochrome genes [34]. It has been suggested that the *c*-type cytochromome (the total of *c*-type cytochromes encoded in a given genome) is largely responsible for the respiratory versatility of these microbes. However, most of *S. oneidensis c*-type cytochromes are missing in the well-characterized model bacteria, such as *E. coli*, it therefore remains a major challenge to annotate their encoding genes with a specific function. Moreover, the focus of studies on *c*-type cytochromes of *S. oneidensis* has been limited to those involved in the anaerobic respiration of various different electron acceptors, especially soluble and insoluble metal oxides [16]. As a result, functions of a large fraction of the entire *c*-type cytochrome pool remain unknown because many of deletion mutants in *c*-type cytochrome genes do not have distinct phenotypes under conditions tested [10,35]. This is, to some extent, attributable to the promiscuity of *c*-type cytochromes given that a large number of proteins sharing similar features coexist in the proteome. For example, terminal reductases OTR and NrfA are able to reduce multiple substrates [12,36]. More importantly, functional redundancy and degeneration of *c*-type cytochromes may be a common scenario in *S. oneidensis*. Three of 44 *c*-type cytochromes predicted initially are either truncated or disrupted, thereby unlikely to be functional [9]. In the case of genes for intact *c*-type cytochromes, approximately 40% of them are transcribed at extremely low levels and/or their products are not identified in proteomes from cells grown under either aerobic or anaerobic condition, among which the *coxB* gene (the *caa*_3_ oxidase component) is verified [17,22]. Therefore, to determine physiological roles of unknown *c*-type cytochromes remains an important task for the future.

## Methods

### Bacterial strains, plasmids, and growth conditions

A list of all bacterial strains and plasmids used in this study is given in Table 2. Information of primers used for PCR amplification in this study is available upon request. For genetic manipulations, *E. coli* and *S. oneidensis* strains were grown in Luria-Bertani (LB, Difco, Detroit, MI) medium at 37 and 30^o^C, respectively. Where required, the growth medium was supplemented with chemical agents at the following concentrations: 2, 6-diaminopimelic acid (DAP), 30 µM; ampicillin, 50 µg/mL; kanamycin, 50 µg/mL; and gentamycin, 15 µg/mL.

**Table 2:** Strains and plasmids used in this study.

Strain or plasmid	Description	Reference or source
*E. coli* strain		
DB3.1λ	Host for pMK2010	23
WM3064	Host for *pir*-dependent plasmids and donor strain for conjugation; Δ*dapA*	W. Metcalf, UIUC
*S. oneidensis* strains		
MR-1	Wild-type	Lab stock
HG0264	As MR-1 plus Δ*scyA*	This study
HG0265	As MR-1 plus Δ*ccmI*	This study
HG0266	As MR-1 plus Δ*ccmF* (*ccmF1*)	This study
HG0268	As MR-1 plus Δ*ccmH*	This study
HG0269	As MR-1 plus Δ*SO0269*	This study
HG0478	As MR-1 plus Δ*ccmF2*	This study
HG0478-6	As MR-1 plus Δ*SO0478 -6*	This study
HG1233	As MR-1 plus Δ*torC*	This study
HG1748	As MR-1 plus Δ*SO1748*	This study
HG2178	As MR-1 plus Δ*ccpA*	This study
HG3056	As MR-1 plus Δ*SO3056*	This study
Plasmids		
pDS3.0	Ap^r^, Gm^r^, derivative from suicide vector pCVD442	24
pMK2010	Donor vector for *ccdB*-Cam^r^ cassette flanked by *attP1* and *attP2*	23
pHGM01	Ap^r^, Gm^r^, Cm^r^, *att*-based suicide vector	This study
pHG101	Promoterless broad-host Km^r^ vector	40
pHG102	pHG101 containing the *S. oneidensis arcA* promoter	40

### qRT-PCR

qRT-PCR analysis of RNA extracted from mid-logarithmic growing cells (OD_600_, ^~^0.4 and ^~^0.15 for aerobic and anaerobic cultures respectively) were carried out with an ABI7300 96-well qRT-PCR system (Applied Biosystems) essentially as described previously [37]. The expression of each gene was determined from three replicas in a single real-time qRT-PCR experiment. The Cycle threshold (*C*_*T*_) values for each gene of interest were averaged and normalized against the *C*_*T*_ value of the *arcA* and *recA* genes, whose abundance was constant during the exponential phase [38,39]. Relative abundance (RA) of each gene was standardized to the *C*_*T*_ values of both the *arcA* and *recA* genes using the equation RA = 2^-∆CT^, yielding similar fold differences.

### Construction of in-frame mutants with a new mutagenesis-construct delivery plasmid employing BP recombination

The fusion PCR method used for construction of *S. oneidensis* in-frame deletion mutants involves with multiple rounds of PCR and conventional cloning, both of which greatly reduce its efficiency and effectiveness [24]. To make an improvement, in this study we developed a new mutagenesis-construct delivery vector employing *attB*-*attP* (BP) recombination based on the system reported before [24]. This plasmid, namely pHGM01, was created by introduction of a DNA fragment containing *attP1*, *ccdB*, Cm^R^, and *attP2* from pMK2010 into the *Sac*I site of pDS3.0 [23,24] (Figure 2A). pHGM01 was propagated in *E. coli* strain DB3.1 λ*pir*, which is able to maintain plasmids possessing a *ccdB* gene and an R6K γ origin of replication [23]. To construct an in-frame deletion mutant using pHGM01, *attB1* and *attB2* sequences were arranged next to gene specific sequences within the 5-O and 3-O primers, which located at each end of the fusion PCR product (in-frame deletion construct) for subsequent site specific recombination, which was performed using the BP Clonase (Invitrogen) according to the manufacturer’s instruction (Figure 2B). The resulting recombination mixture was transformed into *E. coli* strain WM3064, which permits the replication from the R6K γ origin but is sensitive to the *ccdB* gene. After recombination between *attB* and *attP* sequences, the fragment flanked by *attP1* and *attP2* within pHGM01 was replaced by the in-frame deletion construct, removing the *ccdB* gene. As a consequence, resultant destination vectors in which the recombination occurred enabled WM3064 cells to grow on plates containing ampicillin (and/or gentamycin) whereas the *ccdB* gene carried on the pHGM01 was toxic to WM3064. The correct destination vector for mutagenesis, verified by DNA sequencing, was then transferred into *S. oneidensis* by conjugation and the rest of the mutagenesis procedure was carried out the same as described before [24]. The final in-frame deletion mutant was confirmed by sequencing the mutated region.

### Complementation of in-frame deletion mutants

Plasmids pHG101 and pHG102 were used in genetic complementation of mutants [40]. For complementation of genes next to their promoter, a fragment containing the gene of interest and its native promoter was generated by PCR and cloned into pHG101. For the rest genes, the gene of interest was amplified and inserted into MCS of pHG102 under the control of the *arcA* promoter, which is constitutively active [38]. Introduction of each verified complementation vector into the corresponding mutant was done by conjugation, and verified by PCR and restriction enzyme mapping.

### Physiological characterization of mutant strains

For growth measurements under aerobic and anaerobic conditions, M1 defined medium containing 0.02% (w/v) of vitamin-free Casamino Acids was used as described previously [41,42]. Anaerobic media and cultures were prepared as reported earlier [18,41]. Growth of mutant strains was measured using a Bioscreen C microbiology reader (Labsystems Oy, Helsinki, Finland) by recording optical densities of cultures at 600 nm every 15 minutes. Generation times were calculated during the exponential growth phase.

### Biochemical methods

Cells of the mid-exponential phase were harvested and then were lysed with lysis buffer (0.25 M Tris/HCl, (pH 7.5), 0.5% Trion-X100). Protein concentration was determined with a bicinchoninic acid assay kit with bovine serum albumin (BSA) as a standard according to the manufacturer’s instructions (Pierce Chemical). The amount of heme *c* was measured following the procedure described elsewhere [43].

### Statistical analyses

Statistical significance of the difference between experimental groups was assessed by two-way analysis of variance (ANOVA) followed by Bonferroni posttests.
